# Fixation Techniques to Maintain Position for a Cross Leg Flap: Technical Tips and Algorithmic Approach

**DOI:** 10.1055/s-0044-1786194

**Published:** 2024-06-13

**Authors:** Vinita Puri, Raghav Shrotriya, Chandrashekhar Chalwade

**Affiliations:** 1 Department of Plastic Surgery, Seth GS Medical College and KEM Hospital, Mumbai, Maharashtra, India

High-speed road traffic accidents may lead to composite or soft tissue defects in the leg. In cases of severe leg trauma specially in vessel depleted leg, cross leg flap remains the lifeboat solution for wound coverage. With the advent of microsurgical techniques, the frequency and indications of cross leg flaps procedure has reduced. Nevertheless, it is advisable to have cross leg flap in the reconstructive armamentarium to be used in relevant situations as a last resort or in places where the microsurgical expertise is not available. 1

A cross leg flap is one which has its vascular pedicle attached to the uninjured limb while being inset onto the defect on the injured opposite limb. It may be inferiorly based fasciocutaneous flap or a standard (medially based) cross leg flap or a propeller perforator flap or even a free flap 2 with anastomosis done to a pedicle taken from the uninjured limb. In case of a cross leg design, the advantages of the propeller flap may be lost as the distal part of the flap may not cover the donor defect as the turn may be just 90 degrees. The common prerequisite in all these cases is a very stable fixator system to maintain the relative position of the two limbs (to prevent undue tension on the carrying segment or the vascular pedicle). This fixation is crucial for the flap survival as well as for patient comfort in the postoperative period. Fixation may be achieved by Plaster of Paris (PoP) splints or by placing external fixators in the limbs. In this paper, the authors discuss the process of decision-making regarding the type of the fixation technique and some technical tips on fixation. Informed consent was taken from all patients for the purpose of this paper.

**Fig. 1:**
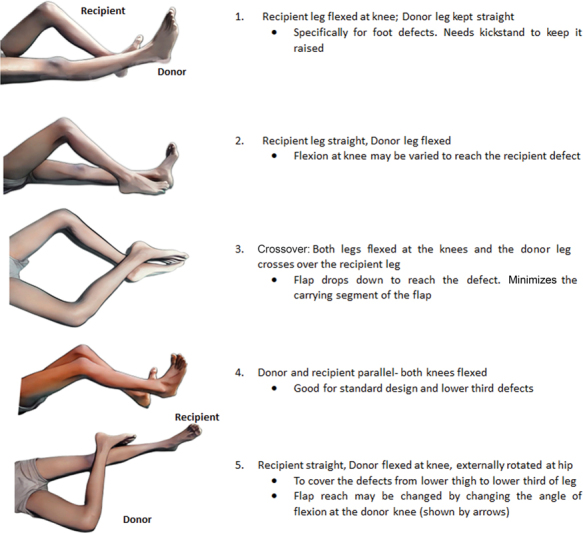
The commonly used positions for performing a cross leg flap. Suitable position must be chosen as per the type of flap and the patient's comfort.

The first step is to “plan-in-reverse.” Placing the flap over the defect, the carrying segment should then be placed on the other (uninjured) limb in the most suitable position. The suitability of the position is decided by the patient's comfort and the ease of the flap to reach the defect in the most efficient manner. The aim is to place the base of the flap close to the defect on the other limb in a comfortable position (to be maintained for 3 weeks). For this purpose, five commonly used limb positions are shown in Fig. 1 . It is important to understand that these are the most commonly used positions and there may be variations of these both in terms of longitudinal and rotational positioning between the two legs as per the requirement of the situation.

**Fig. 2:**
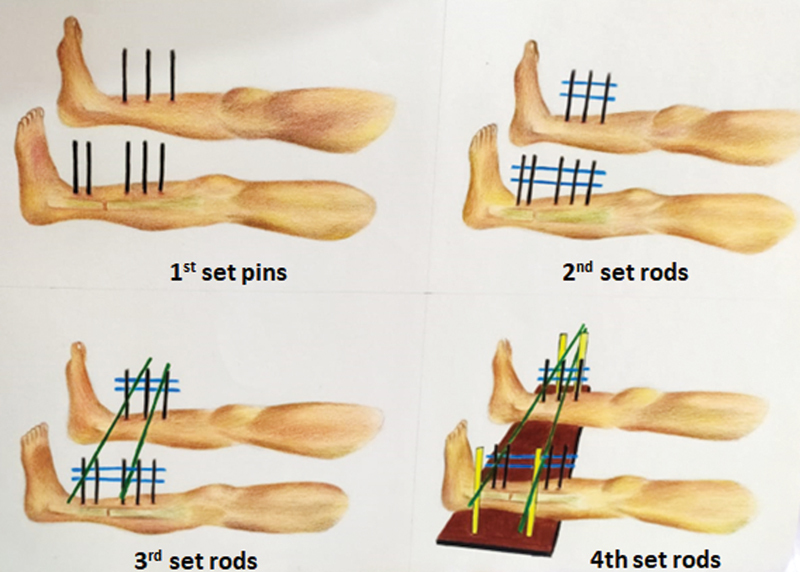
Schematic diagram showing the hierarchy of fixator placement for a cross leg flap. 1st set pins are shown marked in **Black** , 2nd set rods in **Blue** , 3rd set rods in **Green** , and 4th set rods are in **Yellow** ***.***

**Fig. 3:**
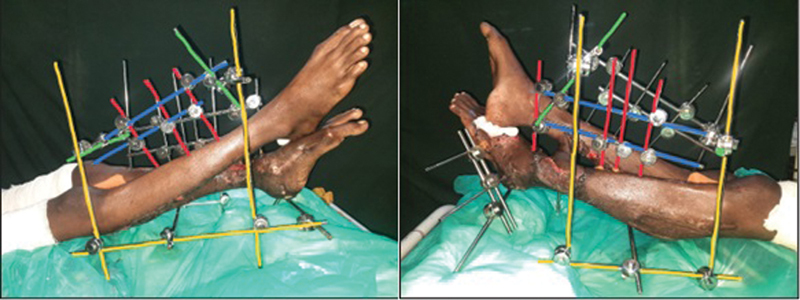
Cross leg frame in a patient showing color coding for 1st, 2nd, 3 ^rd^ , and 4th set of pins is as described in Fig. 2 . Opposite side pins have not been colored for the sake of clarity.

Generally, for an inferiorly based flap, it is advisable to place the donor foot flat on the bed and just adjacent to the recipient leg defect and for a standard design, a parallel or crossover placement may be needed. In case of cross leg free flaps, posterior tibial vessels may be best used for anastomosis and therefore the limb placement should aim at attempting to bring the ankle close to the point of pedicle exit on the recipient limb. Planning should always allow separation of minimum 2 to 3 cm between the legs so that they do not touch and form pressure sores. This is important, as despite the best attempts at rigid fixation, there is always some laxity and there is a chance that the legs may touch each other. The flap is raised according to this plan and secured to the donor limb with a mop and bandage. Now the required position is communicated to the orthopaedic surgeon. Often the fixator placement seems daunting and complicated. The authors propose an algorithmic approach to decide on the type of fixator frame that may be devised. There are four sets of pins used ( Figs. 2 and 3 ):

1st set: 3.5-mm Shantz pins which take purchase in the bone. Generally, three vertical pins are placed through the subcutaneous border of tibia. Three pins proximal to the fracture site and one or two pins distal to the fracture site are placed in the injured limb. These may be placed little obliquely to avoid colliding with the pins in the other limb.
2nd set: connecting rods to stabilize the 1st set pins. Generally, two horizontally placed rods are connected using universal joints.
3rd set: connecting rods to hold the limb in relative position. At least two 3rd set connecting rods are placed between the 2nd set rods of both legs to keep them in desired relative position. These may be vertical, horizontal, or oblique as the position demands.
4th set: Rods form a kickstand providing elevation to the whole construct. One proximal and one distally placed vertical rod on each limb to form the elevated kickstand. Additional horizontal connecting rods may be added below the legs to prevent splaying of the kickstand rods and to increase the stability of the construct ( Fig. 3 ).

**Fig. 4:**
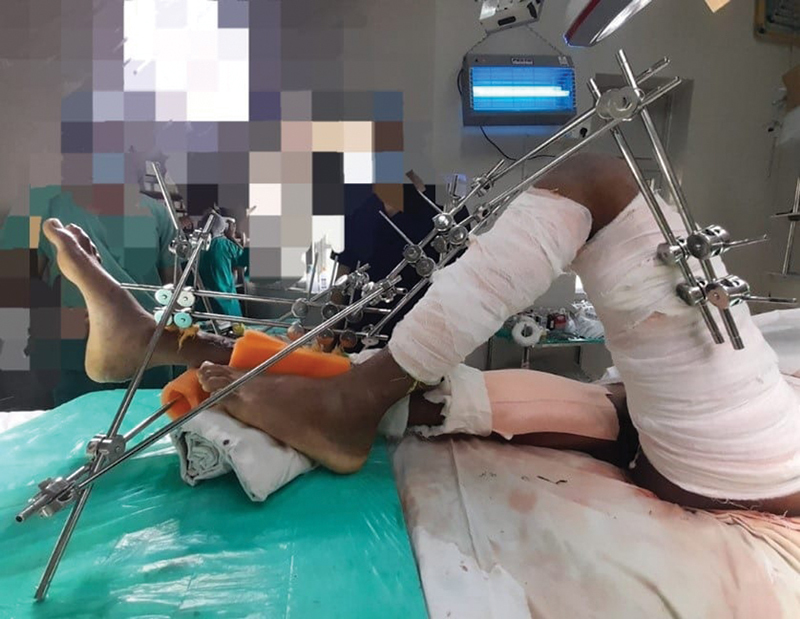
1st and 2nd sets of pins may be placed in the lateral aspect of femur and connected to the tibia pins to maintain the requisite angle in case the knee needs to be kept flexed.

- If the 3rd set rod is horizontally placed, → connects directly to 4th set rod
- If the 3rd set rod is oblique/vertically placed→ connects to a horizontal rod→ connects to 4th set rod
- Positioning may be hindered by the fixator pins in the foot→ frame may need to be modified to accommodate space for the adjacent limb
- If the knee needs to be flexed, additional 1st and 2nd set pins may be placed in the lateral aspect of femur and connected to the tibia pins to maintain the requisite angle as shown in Fig. 4 .
- In case of a free flap, the 1st set pins should be put before anastomosis and rest can be applied after that.

Final insetting of the flap should be done once the legs are properly fixed in position. The sequence of events for a cross leg free flap is as follows: First, Team 1 creates the defect and performs donor vessel dissection on the opposite leg. Following this Team 1 puts the 1st and 2nd set of fixator pins. In the meantime, Team 2 harvests the flap and following this, the anastomosis is done to the donor vessel while the flap is tagged to that limb with temporary sutures. The anastomosis is done with posterior tibial artery (PTA), either as an end-to-end or an end-to-side (ETS) anastomosis. ETS anastomosis may provide a better configuration for the exit of the pedicle towards the recipient limb as a slightly longer pedicle length may be achieved. It is useful to harvest a fascial flap above the PTA to cover the anastomosis as it travels to the other limb. Dressing is done and 3rd and 4th set of pins are now placed to fix the two legs in proper position. Lastly, the flap is inset into the defect.

When the recipient limb has fractures, the 1st set pins are used to stabilize the fracture primarily. In the postoperative period, pin track dressing is given. Both the legs with the frame should be placed over a wide plyboard (and not directly on the wobbly mattress) so as to provide a stable base. Patient should be given a water/air bed and advised position changes to prevent formation of sacral or ischial or scapular pressure sores. Since the fixation is semirigid and slight movement may occur over time, it is important to tighten the link joints on alternate days (as the frame tends to become loose gradually) and to keep air/water-filled gloves or gamjee rolls to maintain the distance between the legs. For doing local flap dressings in between the frame, long forceps may be useful. After 3 weeks, the flap is trained/delayed, which may be done by applying a clamp (if the carrying segment is flat) or by tying circumferentially (if the carrying segment is tubed or in case of a free flap). Irrespective of the type of flap, if the basal inset is adequate to support it, the process of training and division should be started. Once the training is done, flap is divided and the legs may be separated along with removal of pins from the donor limb. This should be done under anesthesia as the joint movements are very painful after such a long period of immobilization. Both limbs should be gradually started on mobilization as stiffness may have occurred due to long immobilization.

**Fig. 5:**
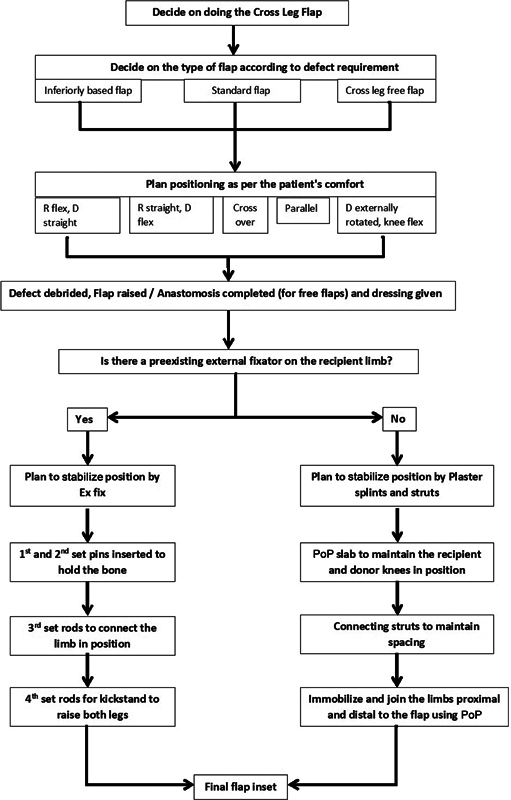
Our algorithm for decision-making while doing a cross leg flap (R, recipient leg; D, donor leg). PoP, Plaster of Paris.

In case the recipient limb does not need fixators for tibia fracture, and the patient does not want anything done to the uninjured limb, the immobilization can be done using PoP splints and connecting struts made by wooden foot scales which are easily available in the market. An algorithmic approach may simplify decision-making, smoothen the execution, optimize the results, and minimize the complications. The authors hereby propose the following algorithm that has been developed in their unit over the last 25 years. ( Fig. 5 ). Over the 25 years, the authors have carried out 47 cross leg flaps. In the first half of this period, the authors mostly did standard design flaps and used plaster splints with wooden scales for stabilization while in the recent decade, the inferiorly based flaps have outnumbered the standard cross leg flaps. Use of external fixators for cross leg flaps has also gradually increased in the recent decade as compared to the PoP fixation because of relatively rigid fixation and postoperative ease of dressings. Eighteen flaps were done as standard cross leg design (medially based) while 19 were done as inferiorly based fasciocutaneous flaps. Ten flaps were free flaps. One patient had a grade 1 pressure ulcer due to inadvertent touching of the feet which healed on revision of position. None of the patients had sacral ulcers and eight patients had heel ulcers which healed well after the flaps were divided. Subjectively, all patients were comfortable and accepted the position of fixation easily since it was decided with their consent after multiple sessions of planning.

In a review article, Mahajan et al 3 have described an algorithm for indications for cross leg flaps in cases of lower limb trauma but they have not discussed about the type of fixators used in their cases. They have preferred the use of superiorly based flap for the knee, conventional flap for upper and middle third defects, and distally based flaps for foot and lower third defects. The authors believe using inferiorly based fasciocutaneous flap (including the sural nerve in the flap) provides higher degree of freedom for the movement of flap to the desired defect while providing a long and narrow carrying segment, and thus are preferred in most cases by the authors, though the choice of flap is flexible and should not be thought of as dogmatic. In their series of 56 patients operated for cross leg flaps, Lu et al 4 train the flap from sixth postoperative day and divide it on an average of 11 days, which is different from this study in that the authors start training from 15th day and divide and inset by 21 days. Garg et al 5 have advocated the use of external fixators for all cases of cross leg flap but have commented on the potential of developing pressure sore over the heel. Alternatively, Lu et al 4 have described the use of circumferential plaster cast for all 56 patients in their series. The authors prefer to stabilize the limbs using external fixator on the donor limb if there is a preexisting fixator on the recipient limb, otherwise both can be stabilized by plaster splints. We have described the use of 4th set of rods ( Fig. 2 ) to form a kickstand on which the limbs are elevated and the incidence of heel pressure sores may be eliminated. Nevertheless, care needs to be taken to prevent pressure sores in other parts of the feet or knees that may be touching or the sacral region by prescribing regular positional offloading and use of water or air mattresses.

On exhaustive literature review, the authors have not found any previous description of the external fixator frame creation specific to the context of cross leg flaps. This article may enhance the reader's understanding of position maintenance in cross leg flaps. The cross leg flap is a valuable tool for the reconstructive surgeons, especially in complex wounds with severe vascular injuries. It requires thoughtful preoperative planning to decide the patient position, type of flap, and the type of stabilization of the limbs. The use of the 4th set of external fixator pins keeps the heels off the bed, prevents pressure ulcers, and allows easy wound care. The algorithm provided may be helpful in optimizing results in these cases.
